# Lactation failure in Src knockout mice is due to impaired secretory activation

**DOI:** 10.1186/1471-213X-8-6

**Published:** 2008-01-23

**Authors:** Harriet Watkin, Monica M Richert, Andrew Lewis, Kristina Terrell, James P McManaman, Steven M Anderson

**Affiliations:** 1 Department of Pathology, University of Colorado Health Sciences Center, Research Complex I, South Tower, Mail Stop 8104, 12801 East 17th Avenue, Aurora, CO 80045, USA; 2 Department of Physiology and Biophysics, University of Colorado Health Sciences Center, Research Complex I, South Tower, Mail Stop 8307, 12801 East 17 th Avenue, Aurora, CO 80045, USA; 3 Department of Obstetrics and Gynecology, University of Colorado Health Sciences Center, Research Complex I, South Tower, Mail Stop 8309, 12801 East 17 th Avenue, Aurora, CO 80045, USA; 4 Department of Pathology, University of Alabama at Birmingham, Volker Hall, G-038A, 1670 University Blvd, Birmingham, AL 35294, USA

## Abstract

**Background:**

Mammary gland development culminates in lactation and is orchestrated by numerous stimuli and signaling pathways. The Src family of nonreceptor tyrosine kinases plays a pivotal role in cell signaling. In order to determine if Src plays a role in mammary gland development we have examined mammary gland development and function during pregnancy and lactation in mice in which expression of Src has been eliminated.

**Results:**

We have characterized a lactation defect in the Src-/- mice which results in the death of over 80% of the litters nursed by Src-/- dams. Mammary gland development during pregnancy appears normal in these mice; however secretory activation does not seem to occur. Serum prolactin levels are normal in Src-/- mice compared to wildtype controls. Expression of the prolactin receptor at both the RNA and protein level was decreased in Src-/- mice following the transition from pregnancy to lactation, as was phosphorylation of STAT5 and expression of milk protein genes. These results suggest that secretory activation, which occurs following parturition, does not occur completely in Src-/- mice. Failed secretory activation results in precocious involution in the mammary glands of Src-/- even when pups were suckling. Involution was accelerated following pup withdrawal perhaps as a result of incomplete secretory activation. In vitro differentiation of mammary epithelial cells from Src-/- mice resulted in diminished production of milk proteins compared to the amount of milk proteins produced by Src+/+ cells, indicating a direct role for Src in regulating the transcription/translation of milk protein genes in mammary epithelial cells.

**Conclusion:**

Src is an essential signaling modulator in mammary gland development as Src-/- mice exhibit a block in secretory activation that results in lactation failure and precocious involution. Src appears to be required for increased expression of the prolactin receptor and successful downstream signaling, and alveolar cell organization.

## Background

The mammary gland is a unique organ in that development primarily takes place postnatally. During puberty, primarily under the influence of estrogen, ductal elongation and branching occurs to form the primary ductal tree [1-3]. At the onset of pregnancy, progesterone and prolactin stimulate the outgrowth of both lateral and alveolar buds resulting in the formation of secretory lobuloalveolar units [4-6]. During lactation these lobuloalveolar units produce and secrete the milk needed to sustain the viability and growth of newborn pups. At parturition there is a dramatic increase in the expression of milk protein genes and lipid biosynthetic enzymes [7,8]. The transition from late pregnancy to lactation is referred to as secretory activation [9,10], and this transition is stimulated by a rise in prolactin (PRL) and decrease in serum progesterone [11-14]. While the molecular mechanisms underlying secretory activation remain obscure, the expression of several signaling molecules and transcription factors increases at this time, including Src (Richert et al, unpublished data), Akt [7,8,15], Elf5 [16], and sterol response element binding protein (SREBP) [7,8]. Each of these factors may play either unique or redundant roles in secretory activation. Copious production of milk during lactation is stimulated by PRL, which induces expression of milk protein genes and maintains viability of the mammary epithelial cells [17-19]. The role of PRL in regulating lipid biosynthesis by mammary epithelial cells is less well established, and is likely to be heavily influenced by the diet of the mouse [8,10]. Upon weaning of the pups, the mammary gland undergoes involution: involution is characterized by the apoptosis of the majority of the mammary epithelial cells and degradation/remodeling of the extracellular matrix by matrix metalloproteases (MMPs) resulting in ductal structures that closely resemble those present in the virgin mouse [20].

Src is the prototype of the Src family of nonreceptor protein tyrosine kinases, a family that includes eight closely related members which share common structural motifs [Src, Fyn, Yes, Fgr, Lyn, Lck, Hck, and Blk] [21]. The activation of this family of protein kinases is regulated by interaction of either the SH2 or SH3 domains of Src family kinases with specific ligands that results in structural changes leading to catalytic activation of the kinase domain [22,23]. Src family kinases can be activated by stimulation of cells with peptide growth factors [24-26], steroid hormones [27-29], and through engagement of integrins by binding to extracellular matrix [30]. Thus Src family kinases appear to be a central node in signaling by numerous receptor complexes. Since mammary gland development and function is regulated by all of these stimuli (peptide growth factors, steroid receptors and integrins), we expected that mammary gland development and/or function might be disrupted in mice lacking expression of Src. It has been observed that mammary gland development during puberty is altered in the Src-/- mice [31] (Richert et al, unpublished data) such that ductal elongation is delayed in Src-/- mice compared to wildtype Src+/+ mice. Mechanisms that may underlie the delay in ductal elongation include changes in estrogen receptor signaling or a change in the amount of estrogen receptor [31], or a requirement for expression of Src in the stromal cell compartment of the mammary gland (Richert et al, unpublished data). While conducting these studies we also observed that Src-/- mice display many aspects of a lactation failure including death of most of the pups and retarded growth of any surviving pups. In this manuscript we report that Src is required for the induction of lactation and Src-/- mice exhibit a block in secretory activation that results in lactation failure, death of newborn pups, and precocious involution.

## Results

### Src deficient mice have normal pregnancy-induced development

**Figure 1:**
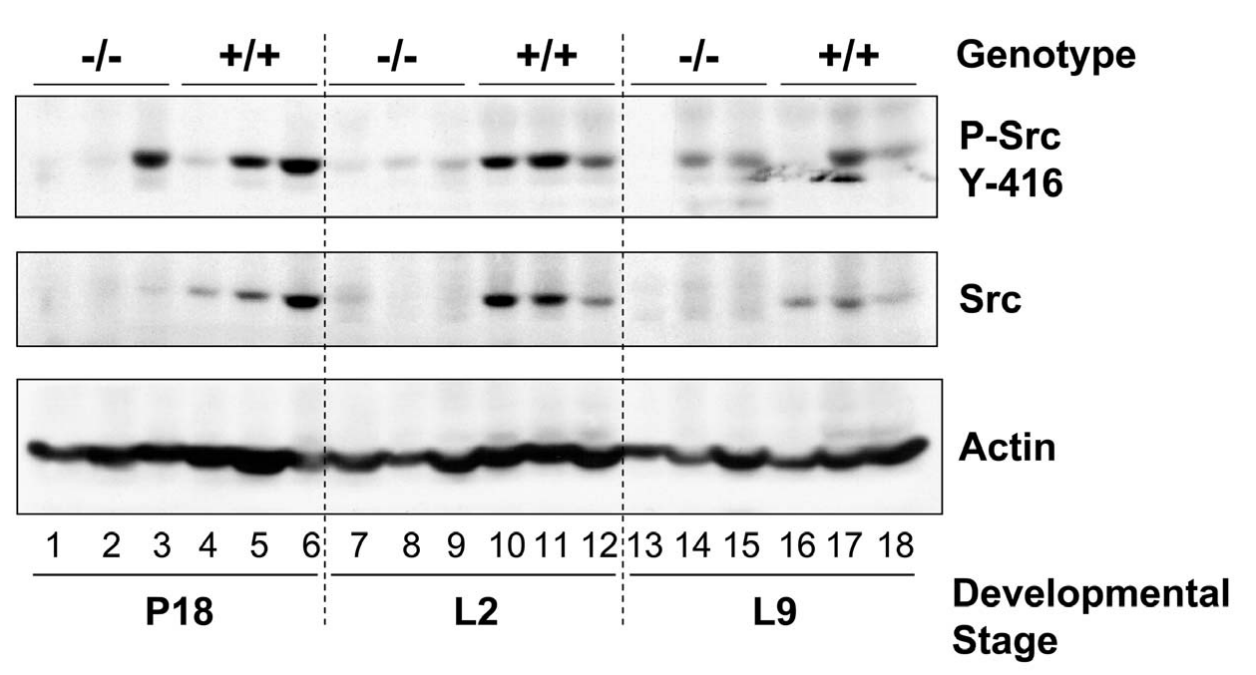
**Src is expressed and activated in wildtype mammary glands during pregnancy and lactation**. The number 4 mammary gland was removed from Src-/- and Src+/+ mice on day 18 of pregnancy (P18) (lanes 1–6), lactation day 2 (L2) (lanes 7–12), and lactation day 9 (L9) (lanes 13–18). Three separate mice were used per genotype and developmental stage. Protein lysates were prepared as described in the Materials and Methods sections, and immunoblotting conducted to detect phosphorylated Src (tyrosine^416^) (top panel), total Src (middle panel), total actin, as a loading control (bottom panel).

Mammary gland development occurs in defined stages that are linked to sexual development and reproduction. Src is required for ductal elongation during pubertal development of the mammary gland [31] (Richert et al, unpublished data). Gene expression analysis and immunoblot analysis demonstrates that expression of Src increases late in pregnancy during the transition from pregnancy to lactation (Richert et al, unpublished data), a transition referred to as secretory activation [9,10,32]. The expression of Src in the mammary gland during pregnancy and lactation was examined by immunoblot analysis of whole mammary gland lysates harvested at day eighteen of pregnancy and days two and nine of lactation (Figure 1). Src protein expression was detected at all three of these stages in lysates prepared from Src+/+ mice, although the amount of Src protein is lower at day nine of lactation compared to day two of lactation. While there is some variation between the three different animals shown at each time point, the general trend is clear; there is an increase in the amount of Src between day eighteen of pregnancy (P18) and day two of lactation (L2), followed by a decrease in the amount of Src protein by day nine of lactation (L9) (Figure 1, middle panel, lanes 4–6, 10–12 and 16–18). No Src was detected in lysates of glands harvested from Src-/- mice (Figure 1, middle panel, lanes 1–3, 7–9 and 13–15). To examine the catalytic activation of Src, protein lysates were immunoblotted with an anti-phospho-Src antibody that specifically detects Src family members when phosphorylated on tyrosine^416 ^in the activation loop of the kinase (Figure 1, top panel). Phosphorylation of tyrosine^416^, which correlates with the catalytic activation of Src, was apparent in lysates prepared from the mammary glands of wildtype mice at P18, L2, and L9 (Figure 1, top panel, lanes 4–6, 10–12 and 16–18). Although most of the lysates isolated from Src-/- mice do not appear to react with the anti-phospho-Src tyrosine^416 ^antibody, immunoreactive material is detected in several of the samples (Figure 1, top panel, lanes 3, 14 and 15). The anti-phospho- Src tyrosine^416 ^antibody is not specific for Src and is able to recognize the same phosphorylation site in the activation loop in Fyn, Lyn, Lck, Yes, and Hck; of these Src family members only Fyn is expressed in high levels in the mammary gland (Richert et al, unpublished data) [33]. Therefore we believe that this band represents activated Fyn in these samples, although we have not directly demonstrated this by immunoprecipitation of Fyn from these samples. Each lane represents tissue lysates harvested from different animals, and it would be expected that there might be animal-to-animal variations in the expression and activation of Src. The fact that Src is expressed and is activated during late pregnancy and lactation stimulated us to determine whether it plays a role in the complex signaling events that occur within the mammary epithelial cell during this developmental phase.

**Figure 2:**
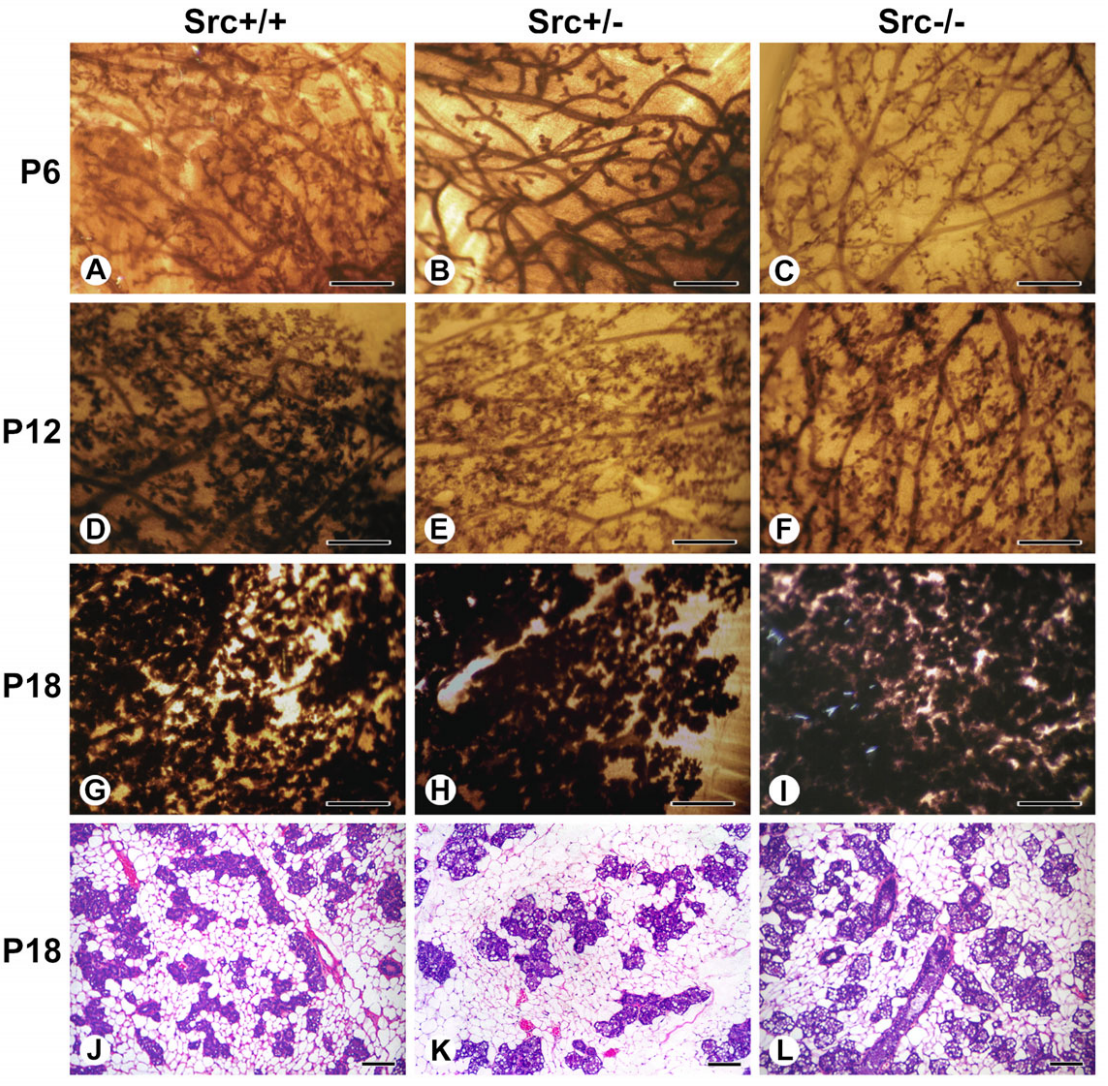
**Src deficient mice have normal pregnancy-induced development**. Whole mounts of the number 2 and 3 mammary gland of Src+/+ (A, D, G, and J), Src+/- (B, E, H and K) and Src-/- (C, F, I and L) mice at P6 (A-C), P12 (D-F) and P18 (G-I). Histological sections from mammary glands at P18 are also shown for each genotype (J-L). In panels A to I scale bars represent 5 mm. In panels J to L scale bars represent 100 μm.

Mammary gland development during pregnancy is characterized by the formation of secondary branches and extensive lobuloalveolar development, and results in a dramatic increase in the number of mammary epithelial cells in the mammary gland; based upon the expression levels of keratin-19 and claudin 7, there is a 1000-fold increase in the number of epithelial cells by the end of pregnancy, although when the volume of epithelial cells is considered, a 125-fold increase might be considered to be a more conservative estimate [34]. This phase of mammary gland development is primarily driven by progesterone [6,35]. Whole mounts of mammary glands from Src knockout animals from days six (P6), twelve (P12) and eighteen (P18) of pregnancy demonstrate that at P6 the density of ducts, the extent of ductal branching and alveolar bud formation in Src-/- mice was reduced compared to both Src+/+ and Src+/- mice (Figure 2, panel C compared to A and B). By P18, however, there appeared to be no difference in mammary gland development between Src+/+, Src+/-, and Src-/- mice (Figure 2, panels G, H and I). The density of the epithelial structures in the Src-/- mice appeared similar to glands from Src+/+ mice on day eighteen of pregnancy (Figure 2, panels G and I). Thus the whole mounts indicate that by the end of pregnancy ductal density and lobuloalveolar development in the Src-/- mice is normal (Figure 2, panels G-I). Examination of hematoxylin and eosin stained thin sections of mammary glands isolated from Src+/+, Src+/- and Src-/- mice on P18 also demonstrate that pregnancy-induced development in the Src-/- mice is normal, with prominent densely-staining secretory alveoli surrounded by adipocytes visualized in all three genotypes examined (Figure 2, panels J, K and L). Therefore we conclude that the diminished ductal branching and density observed at P6 may reflect the reduced ductal density and delayed mammary gland development observed in 10 week-old virgin Src-/- mice [31] (Richert et al, unpublished data), as mice of this age were used in these studies, rather than decreased proliferation of mammary epithelial cells during pregnancy.

### Pups suckled by Src knockout mice have significantly reduced weight gain

**Figure 3:**
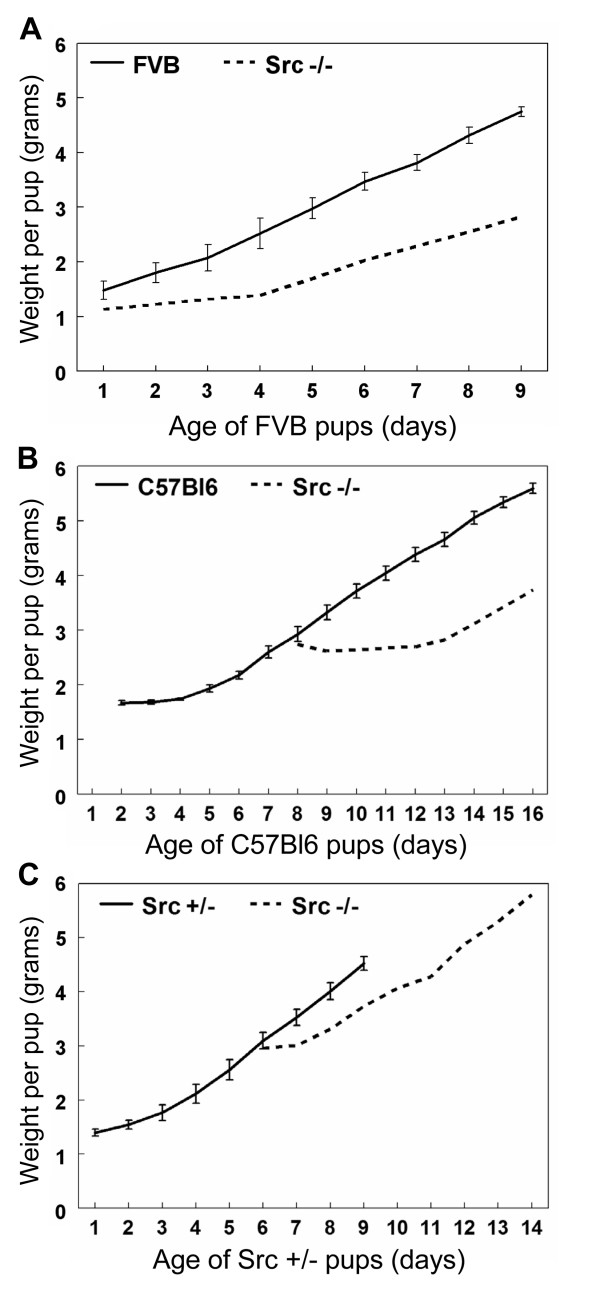
**Pups suckled by Src knockout mice have dramatically reduced weight gain**. Graphs demonstrating the weight gain of pups foster nursed by Src-/- mice compared to the weight gain of pups nursed by control dams. Litters were normalized to eight pups. N = 3 for the control mice and error bars depict the s.e.m. in the growth of these control litters. The start of each graph represents day 1 of lactation (first day post-partum) for the nursing mother. The weights of pups nursed by Src-/- mice were recorded for 9 days. A) Weight gain of a single litter of eight newborn FVB pups fostered by Src-/- mouse in comparison to three litters of FVB pups nursed by FVB dams. B) Weight gain of a single litter of eight C57Bl6 pups that were 8 days old when they were cross-fostered onto a Src-/- mouse that was one day post-partum. The growth rate is compared to three litters of C57Bl6 pups nursed by control C57Bl6 dams. C) Weight gain by a single litter of 6-day old Src+/- pups cross-fostered onto a Src-/- mouse that was one day post-partum in comparison to three litters nursed by Src+/- dams.

Secretory activation occurs following parturition and leads to the production of copious amounts of milk required to nourish pups. The first indication that Src is an important mediator of mammary gland development was observed when the Src knockout mice were unable to support growth and survival of their pups. The litters born to Src-/- dams were small and over 90% of the pups died within three days. In order to assess the apparent lactation failure of the Src-/- mice, average pup weights during the first 9 days of lactation were measured. The pups from the Src-/- mice were replaced with eight newborn pups from wildtype FVB, C57Bl6, or Src+/- mothers (Figure 3, panels A, B and C, respectively). Due to the poor growth and recurrent death of these foster pups, resulting in the death of over 80% of the fostered litters, we also tried replacing the pups of the Src-/- dams with older pups that were "experienced" at nursing. In the course of the experiments to characterize the lactation potential of the Src-/- dams only three fostered litters nursed by the Src-/- dams survived. The pups in each of these three litters had reduced weight gain over the nine days of lactation analyzed compared to litters nursed by control mothers (Figure 3). In Figure 3A eight newborn FVB pups were nursed by a Src-/- mouse. For the first few days the FVB pups showed very little weight gain then after day 4 their weight steadily increased and after nine days of lactation the average pup weight was 2.8 grams, which is much reduced compared to the 4.7 grams for FVB pups nursed by an FVB mother (Figure 3A). When eight day old C57Bl6 pups were cross-fostered onto a Src-/- mouse, in Figure 3B, the growth of these pups was severely diminished compared to those nursed by control dams (Figure 3B). Indeed on day 16, when the cross-fostered pups had been nursed by the Src-/- mouse for 9 days, the average pup weight was 3.7 grams compared to 5.8 grams for the control C57Bl6 pups nursed by a C57Bl6 dam. The 6 day old pups from a Src+/- litter that were fostered by the Src-/- mouse in Figure 3C demonstrated the least detrimental effect on their growth. When they were 9 days old (day 4 of lactation for the Src-/- mouse) the average pup weight was 3.7 grams compared to 4.5 grams for those nursed by Src+/- dams. For each of these studies the growth of pups nursed by Src-/- dams was compared to 3 litters of pups nursed by control dams (FVB, C57Bl6 and Src+/-). Due to the high mortality of pups nursed by Src-/- dams there were no additional litters to include in this analysis. These results demonstrate that Src knockout mice have diminished lactation capacity regardless of the strain or genotype of the pups nursed and therefore support the conclusion that Src is essential for secretory activation and/or lactation.

### Altered mammary gland histology during lactation suggests lactation failure in Src-/- mice

**Figure 4:**
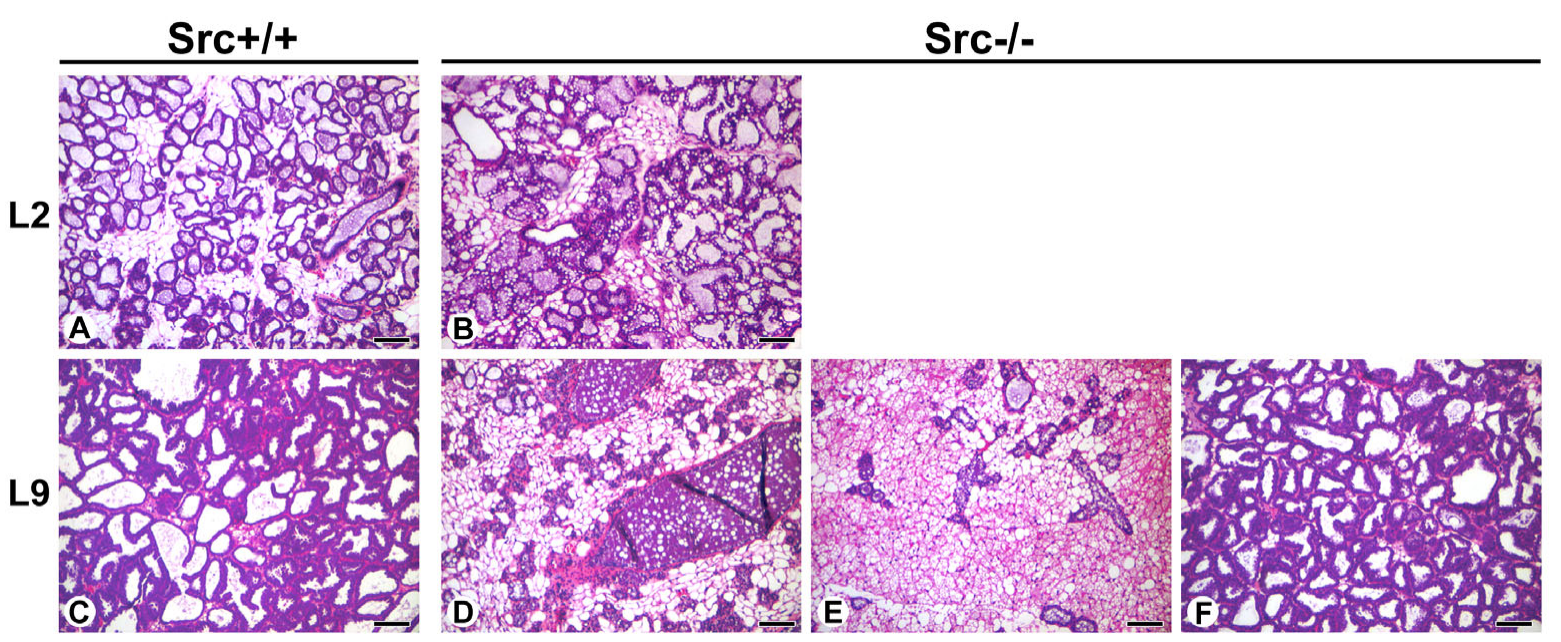
**Altered mammary gland histology during lactation suggests lactation failure in Src-/- mice**. Histological sections from the mammary glands of Src+/+ (A and C) and Src-/- (B and D-F) at L2 (A-B), and L9 (C-F). Three sections of mammary glands from Src-/-mice are shown at L9 (D-F). Scale bars represent 100 μm.

**Figure 5:**
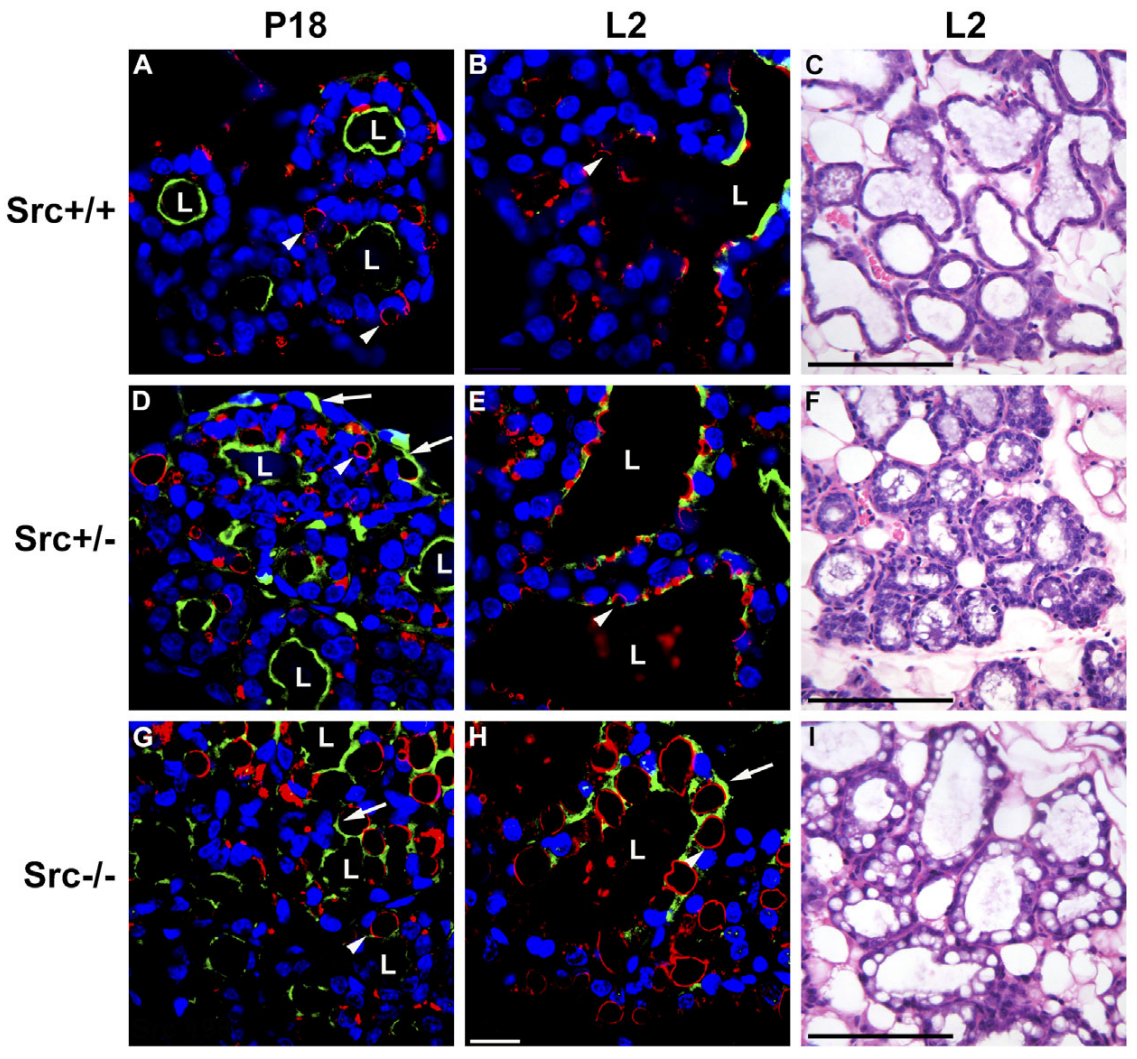
**Secretory activation does not occur in Src-/- mice following parturition**. Histological sections were prepared from the mammary glands of Src+/+ (A-C), Src+/- (D-F), and Src-/- (G-I) mice at P18 (A, D, and G) and L2 (B, C, E, F, H and I). The sections shown in A, B, D, E, G and H were stained anti-ADRP antibody to outline the cytoplasmic lipid droplets (red), Oregon-Green 488-conjugated WPA to outline the surface of secretory alveoli (green), and DAPI to stain the nuclei of mammary epithelial cells (blue). Hematoxylin and eosin-stained sections of mammary glands at L2 are shown in C, F, and I. The bar in panel H represents 10 μm and the bars in panels C, F and I represent 100 μm. Luminal space is indicated by the letter "L", the white arrowheads indicate cytoplasmic lipid droplets, and the white arrows indicates regions where staining with WGA represents atypical patterns not observed in wildtype mice. The black arrows in panel I indicate large cytoplasmic lipid droplets not observed in wildtype mice.

As described above, the mammary glands of Src-/- mice appeared similar to those of Src+/+ and Src+/- mice at P18 with prominent densely-staining secretory alveoli surrounded by adipocytes. By day 2 of lactation (L2) the luminal space of the alveoli from Src+/+ and Src-/- mice had expanded and are clearly evident due to the layer of densely staining mammary epithelial cells that surround the lumen (Figure 4, panels A and B). The mammary epithelial cells of the Src-/- mice have an abundance of large lipid droplets that are not present in mammary epithelial cells from Src+/+ or Src+/- mice, this observation was further investigated (See Figure 5 and below).

By L9 the normal mammary gland is producing copious amounts of milk, approximately 4.5–5 ml/day/dam [36]. The histology of the mammary gland at this time point reflects the expansion of lactation and it is generally characterized by the presence of fully expanded lumens surrounded by a densely staining layer of mammary epithelial cells [37]. The mammary glands of both the Src+/+ and Src+/- mice were similar and displayed the normal morphology expected in lactating mice at this time point [37] (Figure 4, panel C and data not shown). In contrast two different morphologies were observed in the mammary glands of Src-/- mice; in one of the three mice shown in Figure 4, the structure of the mammary gland appeared to be similar to that seen in wildtype (Figure 4), however the mammary glands of the two other Src-/- mice have the morphology expected of a mammary gland that is undergoing involution (Figure 4, panels D and E). In both of these mammary glands the secretory alveoli have collapsed (Figure 4, panels D and E), and a large distended duct that is filled with milk proteins, lipid droplets and apoptotic cells is readily apparent in one of the mice examined (Figure 4, panel D). When compared to the histology of an involuting mammary gland from normal mice, the morphology observed in Figure 4, panel D is typical of what would be expected on day four of involution, while the morphology observed in Figure 4, panel E is typical of a mammary gland on day six to eight of involution when extensive remodeling of the gland has occurred [37]. In all of the cases shown in panels D-F of Figure 4, the dams were nursing pups and thus were considered to be "lactating". In the course of our experiments we have observed that one out of six Src-/- mice exhibits histology similar to that observed in the mammary gland of a wildtype gland at lactation day 9. The mammary glands of the five remaining Src-/- mice displayed histology typically seen in mammary glands undergoing involution, and the morphology observed varied from that normally seen on day two of involution to that seen on day eight of involution. Our observation that one of six Src-/- mice has a normal histology at L9 is consistent with the observation that 3 out 16 mice were able to sustain growth of foster pups over this time period, although at a reduced rate (Figure 3).

### Secretory activation does not occur in Src-/- mice following parturition

The decrease in the concentration of progesterone that occurs immediately prior to parturition combined with the increased concentration of PRL that occurs late in pregnancy, and the PRL that is released by the pituitary in response to suckling, are required for secretory activation [14,38]. Characteristically mammary epithelial cells from late pregnant mice have large cytoplasmic lipid droplets (CLDs) that are often centrally located within the cell [37]. Following parturition only small lipids droplets are observed at the luminal surface of mammary epithelial cells; a change that is thought to reflect secretory activation of cells in the alveolus [10] (T. Russell and J.L. McManaman, unpublished data). At L2 mammary epithelial cells from Src+/+, and Src+/- mice have small lipid droplets at their apical surface (Figure 5, C, and 5F), while mammary epithelial cells from Src-/- mice have large, centrally located lipid droplets (Figure 5I). To further examine the induction of secretory activation following parturition, sections of mammary gland from Src+/+, Src+/- and Src-/- mice were stained with rabbit anti-adipocyte differentiation-regulated protein, also known as adipophilin (ADPH), which is present in the outer membrane of cytoplasmic lipid droplets (CLDs) The luminal surfaces of secretory alveoli were visualized with Oregon-Green 488-conjugated wheat germ agglutinin (WGA), and the nuclei were stained with DAPI (Figure 5, A, B, D, E, G and 5H). Secretory alveoli of Src+/+ mice on day eighteen of pregnancy were readily apparent by the circle of DAPI-stained nuclei deposited around the luminal surface of the alveolus, which was stained with WGA (Figure 5A, luminal space is labeled L). Mammary epithelial cells from Src+/+ mice at P18 contained large lipid droplets that are outlined red by ADRP staining (Figure 5A, white arrowheads). By L2 the luminal space has expanded considerably and the lipid droplets are smaller in size and primarily deposited along the luminal surface of the cells (Figure 5B, white arrowhead). Staining of P18 mammary glands from Src-/- dams in an identical manner revealed poorly organized secretory alveoli with disordered lumen as evidenced by the unorganized pattern of staining with WGA (Figure 5G, white arrow). Large CLDs were apparent in Src-/- mammary gland sections at P18 based upon anti-APDH staining (Figure 5G, white arrowhead). Luminal spaces were more evident when the structure of the secretory alveoli from Src-/- mice at L2 were examined, however, some mammary epithelial cells stained on multiple surfaces with WGA and these cells contained large CLDs (Figure 5H). These observations suggest that Src is required for the structural organization of mammary epithelial cells into secretory lobuloalveolar units. The lack of Src expression results in large CLDs persisting in the mammary epithelial cells at L2, indicating that secretory activation has not occurred. Mammary glands from Src+/- mice have an intermediate phenotype at P18. Organized lobuloalveolar structures are apparent, however, unorganized alveoli are also evident, as are alveoli with WGA staining between cells (Figure 5D, white arrows). By L2, the mammary gland structure in the Src+/- mice appeared to be largely normal with expanded luminal spaces and small lipid droplets (Figure 5E). This indicates that although lobuloalveolar development may be somewhat disorganized in Src+/- mice late in pregnancy, secretory activation post-partum occurs normally and that by L2, the mammary gland exhibits the normal structure of a lactating mammary gland.

### Serum prolactin levels are not altered in Src-/- mice, however expression of the prolactin receptor is diminished

**Figure 6:**
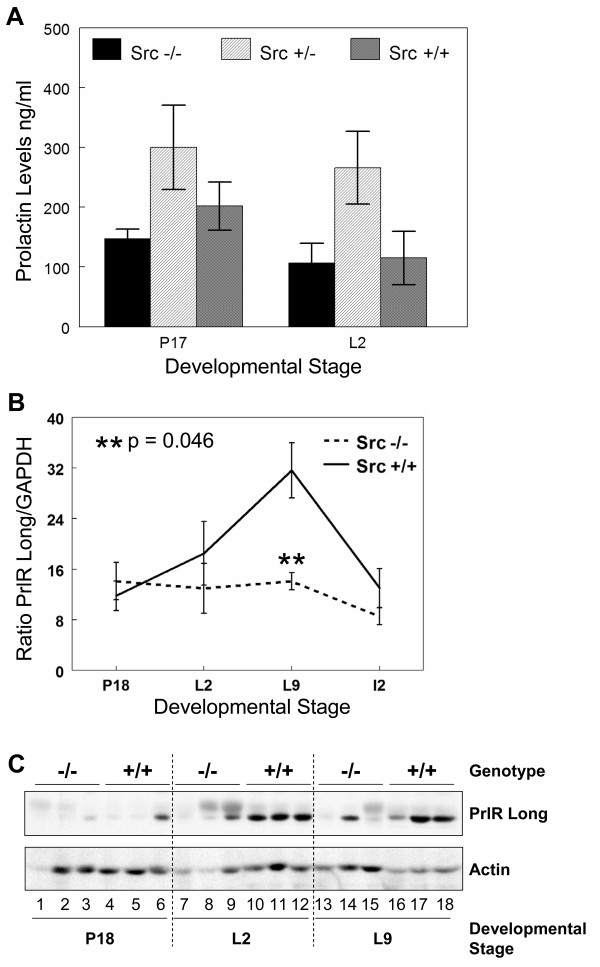
**Serum prolactin levels are normal in Src knockout mice however, prolactin receptor expression is reduced**. A) Blood was drawn from Src-/-, Src+/- and Src+/+ mice at P17 and L2. Prolactin levels were measured using a radioimmune assay and the amount of PRL plotted as the mean (± SEM) of 5 mice for each genotype at the indicated developmental stages. Welch's *t *test was used to evaluate the statistical significance (defined as *P *< 0.05). Src-/- to Src+/+ at P17 *P *= 0.26, Src+/- to Src+/+ at P17 *P *= 0.27. Src-/- to Src+/+ at L2 *P *= 0.89, Src+/- to Src+/+ at L2 *P *= 0.08. B) Total RNA was isolated from the number 4 mammary gland of wildtype and knockout mice at P18, L2, L9, and I 2; three mice were used per genotype and developmental stage. cDNA was synthesized from 1 μg of total RNA and quantitative RT-PCR was performed using primers and probe specific for the long isoform of the prolactin receptor. PRLR message levels were normalized to GAPDH for each sample and the graph represents the mean (± SEM) relative amount of the triplicate tissue samples. C) The number 4 mammary gland was removed from Src-/- and Src+/+ mice at P18 (lanes 1–6), L 2 (lanes 7–12) and L 9 (lanes 13–18). Three separate mice were used per genotype and developmental stage. Protein lysates were prepared as described in the Materials and Methods sections, and immunoblotting conducted to detect the total amount of PRLR expression (top panel), and the amount of actin, as a loading control (bottom panel).

The hormone PRL was first identified by its ability to stimulate mammary gland development and lactation in rabbits and it has since been demonstrated as an essential modulator of proliferation and differentiation of alveoli during pregnancy and milk protein expression during lactation [39-41]. Due to the critical role of PRL in lactogenesis, the amount of serum PRL in Src+/+, Src+/- and Src-/- mice was quantitated to determine whether a reduction in serum PRL could account for the lactation failure in Src-/- mice. Serum PRL was determined in five Src+/+, Src+/- and Src-/- mice at P17 and L2 and were found to be comparable in both the Src+/+ and Src-/- mice (Figure 6A) thus eliminating diminished serum PRL as the mechanism underlying the observed lactation failure in Src-/- mice. It is interesting to note that serum PRL levels were elevated in the Src+/- mice at both times examined, however the increase is not significant.

Since serum PRL levels in the Src-/- mice were similar to those detected in Src+/+ mice we then pursued the hypothesis that the phenotype of the Src-/- mice may result from a block in PRL action. PRL action requires binding to its cognate receptor, the PRL receptor (PRLR). To determine if expression of the PRLR is altered in the Src-/- mice, PRLR levels were examined by quantitative RT-PCR and immunoblotting with an anti-PRLR antibody. The message for the long isoform of the PRLR increased in the mammary glands of wild type mice during lactation, peaked at L9, and then declined by day two of involution. This pattern of expression for the long form of the PRLR during mammary gland development is consistent with previous data [7,8,42]. However, no increase in the levels of mRNA encoding the long form of the PRLR was detected in the Src-/- mice following parturition (Figure 6B). The level of PRLR mRNA in the mammary gland of Src-/- mice remained level from P17 through both lactation time points, and then declined at the second day of involution (I2).

The protein level of PRLR was also increased in Src+/+ mice during lactation compared to the amount of PRLR protein present at P18 (Figure 6C, top panel, lanes 10–12 and 16–18 compared to 4–6). PRLR protein expression was only detected in two of the mammary gland lysates from Src-/- mice (Figure 6C, top panel, lanes 9 and 14). While two of the protein lysates from Src-/- mice at L2 are under loaded as indicated by actin loading control (Figure 6C, bottom panel, lanes 7 and 8), analysis of the third sample suggests that there are lower levels of PRLR protein at this time point, compared to the samples from Src+/+ mice (Figure 6C, top panel, lane 9 versus 10–12). No PRLR protein was detected in two of the three Src knockout lysates from mice at L9 (Figure 6C, top panel, lanes 13 and 15), while the third Src-/- sample shows diminished PRLR levels compared to the samples from Src+/+ mice when then amount of actin present in each sample in taken into consideration (Figure 6C, top and bottom panels, lane 14 compared to 16–18). Thus, although there is some animal to animal variation, we conclude that both PRLR mRNA and protein levels do not increase in Src-/- mice following parturition. The fact that PRLR mRNA does not increase following parturition provides further evidence that secretory activation is altered in Src-/- mice, as expression of the long form of the PRLR has been shown to dramatically increase during this transition [7,8,42].

### Prolactin-dependent signaling pathway is diminished in Src-/- mice

**Figure 7:**
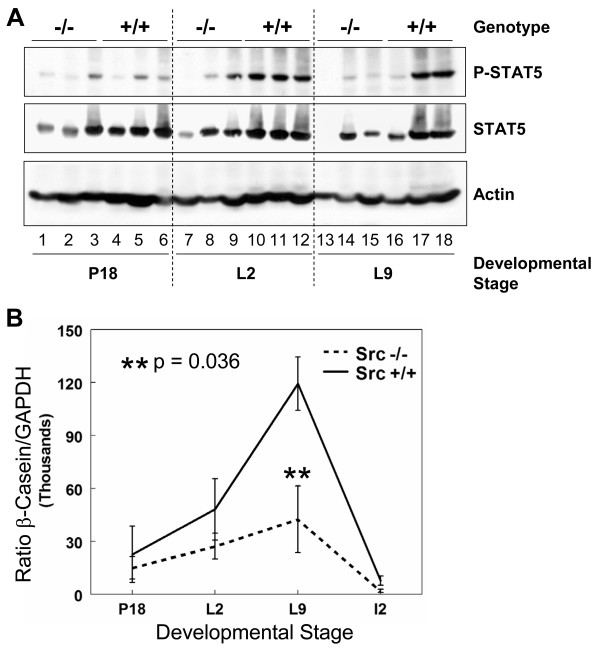
**Downstream signaling from the prolactin receptor is diminished in Src-/- mice**. A) The number 4 mammary gland was removed from Src-/- and Src+/+ mice on day 18 of pregnancy (lanes 1–6), lactation day 2 (lanes 7–12) and lactation day 9 (lanes 13–18). Three separate mice were used per genotype and developmental stage. Protein lysates were prepared as described in the Materials and Methods sections, and immunoblotting conducted to detect phosphorylation of STAT5 (top panel), the amount of total STAT5 (middle panel), and the amount of actin, as a loading control (bottom panel). B) Total RNA was isolated from the number 4 mammary gland of wildtype and knockout mice on day 18 of pregnancy, lactation days 2 and 9 and involution day 2, three mice per genotype and developmental stage were used. cDNA was synthesized from 1 μg of total RNA and quantitative RT-PCR was performed using primers and probe specific for β-casein. β-casein message levels were normalized to GAPDH for each sample and the graph represents the mean (± SEM) relative amount of the triplicate tissue samples.

Downstream components of the prolactin signaling pathway have also been found to be critical for proliferation and differentiation of alveoli during pregnancy and for lactation. Transcription of milk protein genes requires prolactin-mediated activation of signal transducer and activation of transcription (STAT)-5 [18,43-45]. Two isoforms of STAT5 are known to be expressed in the mammary gland; STAT5a and STAT5b, and both isoforms are activated by prolactin in the mammary gland and are able to partially compensate for each other [44]. The levels of STAT5 are not reported to vary greatly during mammary gland development, although the tyrosine phosphorylation of STAT5 increases late in pregnancy and is maintained throughout lactation [46]. The phosphorylation of STAT5 was examined in the mammary glands of Src+/+ and Src-/- mice at P18, L2, and L9, three mice for each timepoint, by immunoblotting mammary gland lysates with anti-phospho-STAT5 (tyrosine^694^) antibody and anti-STAT5 antibody (Figure 7A). The phosphorylation of STAT5 was detected at P18 in Src+/+ mice, and phosphorylation increased further during lactation; high levels of phosphorylated STAT5 were observed in all three mice at L2, and in two of the three samples analyzed at L9 (Figure 7A, top panel, lanes 10–12 and 17–18). Based upon the actin loading control, the total levels of STAT5 in Src+/+ mice did not vary greatly between pregnancy and lactation (Figure 7A, middle and bottom panels, lanes 4–6, 10–12 and 16–18). Phosphorylation of STAT5 in the Src-/- mice at L2 and L9 was dramatically reduced compared to wildtype mice. At P18 the phosphorylation of STAT5 was evident in only one of the three Src-/- samples at a level comparable to that detected in the Src+/+ mice (Figure 7A lane 3 compared to 5–6). During lactation only one of the analyzed Src-/- samples displayed more than a trace level of phosphorylated STAT5 signal (Figure 7A lane 9). The diminished STAT5 tyrosine phosphorylation during lactation provides further evidence that prolactin signaling is impaired in Src-/- mice.

The milk protein β-casein is a transcriptional target of STAT5 downstream of prolactin receptor signaling. In order to determine if the diminished activation of STAT5 detected in the protein lysates from the Src-/- mice had a functional consequence upon the expression of milk protein genes, the levels of β-casein RNA were determined by quantitative RT-PCR. There was a four-fold increase in the amount of β-casein RNA between P18 and L9 in the control mice which is consistent with our findings in others strains of mice [8,10]. There was an approximate two-fold increase in the amount of β-casein RNA in the Src-/- mice between P18 and L9, however, there was as least three-fold less β-casein RNA in the Src-/- at L9 compared to the Src+/+ mice (Figure 7B). The amount of β-casein RNA decreased dramatically with the onset of involution induced by pup withdrawal.

### Src is required for maximal STAT5 activation and β-casein expression in cultured primary mammary epithelial cells

**Figure 8:**
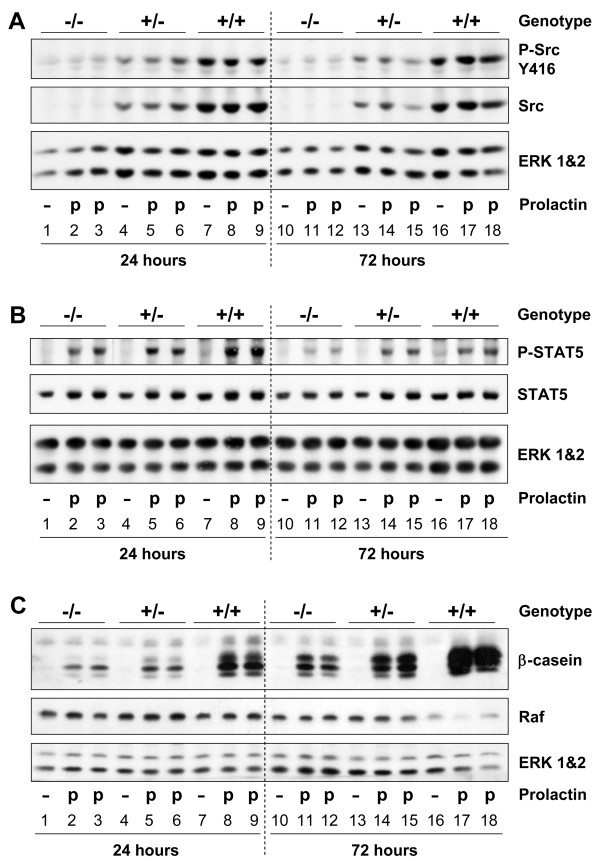
**Primary mammary epithelial cell cultures confirm that Src is required for maximal prolactin signaling**. Primary mammary epithelial cells isolated from Src-/- (lanes 1–3 and 10–12), Src+/- (lanes 4–6 and 13–15) and Src+/+ (lanes 7–9 and 16–18) mice were cultured on Matrigel in growth medium for 3 days then the medium was changed to differentiation medium and the cells were cultured for a further 24 or 72 hours in the presence or absence of prolactin. Cellular proteins were extracted by lysis in NET buffer. A) Immunoblotting was performed to detect phosphorylation of Src (tyrosine^416^) (top panel), total levels of Src protein (middle panel) and the amount of ERK 1&2 (bottom panel) as a loading control. B) Immunoblotting was performed to detect phosphorylation of STAT5 (tyrosine^694^) (top panel), total levels of STAT5 (middle panel) and the amount of ERK 1&2 (bottom panel) as a loading control. C) Immunoblotting was performed to detect β-casein expression (top panel), the levels of Raf and the amount of ERK 1&2 as loading controls (middle and bottom panels).

To determine whether Src was required for PRL-induced activation of STAT5 and expression of β-casein we utilized primary mammary epithelial cells (MECs) isolated from the mammary glands of Src -/-, Src +/-, and Src +/+ mice. MECs were cultured on a laminin-rich basement membrane in the absence or presence of PRL, and protein lysates were harvested after 24 and 72 hours. The expression and activation of Src in the primary MECs isolated from each of the three genotypes was determined by western blotting. Src protein was detected in Src+/+ MECs, and a reduced level was detected in the Src+/- MECs, indicating that there was a dose-dependent relationship between the number of Src alleles and the level of Src protein (Figure 8A, middle panel, lanes 4–9 and 13–18). No Src protein was detected in MECs from Src-/- mice (Figure 8A, middle panel, lanes 1–3 and 10–12). The activation of Src was examined by anti-phospho-Src (tyrosine^416^) antibody and the amount of phosphorylated/activated Src was found to parallel the amount of Src and not to be dependent upon PRL stimulation (Figure 8A, top panel, lanes 4–9 and 13–18). We hypothesize that PRL-dependent Src activation could not be detected in the Src+/+ and Src+/- MECs due to the high basal levels of Src phosphorylation that may result from activation of integrin signaling stimulated by attachment to the laminin-rich basement membrane and the presence of insulin in the differentiation medium. Very little immunoreactive protein was detected with the anti-phospho-Src antibody in Src-/- MECs (Figure 8A, top panel, lanes 1–3 and 10–12), which contrasted with the results obtained in studies of whole mammary gland lysates from Src-/- mice (Figure 2).

PRL-dependent phosphorylation/activation of STAT5 was detected in the MECs isolated from each of the three genotypes of mice (Figure 8B, top panel, lanes 1–18). The level of phosphorylated STAT5 detected in Src-/- MECs was diminished compared to that observed in wild type MECs 24 hours following PRL stimulation, and this decrease was even more evident after 72 hours of stimulation with PRL (Figure 8B, top panel, lanes 2–3 compared to 8–9 and lanes 11–12 compared to 17–18, respectively). The amount of β-casein protein expressed by Src-/- MECs was dramatically reduced compared to the level present in Src+/+ MECs following both 24 and 72 hours of differentiation with PRL (Figure 8C, top panel, lanes 2–3 compared to 8–9 and lanes 11–12 compared to 17–18). The levels of Raf and ERK 1/2 were used to demonstrate equal loading of samples. The level of β-casein protein in MECs isolated from Src+/- mice was intermediate, possibly reflecting a dose-dependent affect of Src upon either the transcription or translation of β-casein (Figure 8C, top panel, lanes 5–6 and 14–15). These data clearly demonstrate that Src has a primary role in PRL-dependent expression of milk protein genes such as β-casein in primary MECs, and demonstrate that this effect can be separated from possible systemic effects of Src in vivo.

### Involution is accelerated in Src-/- mice

**Figure 9:**
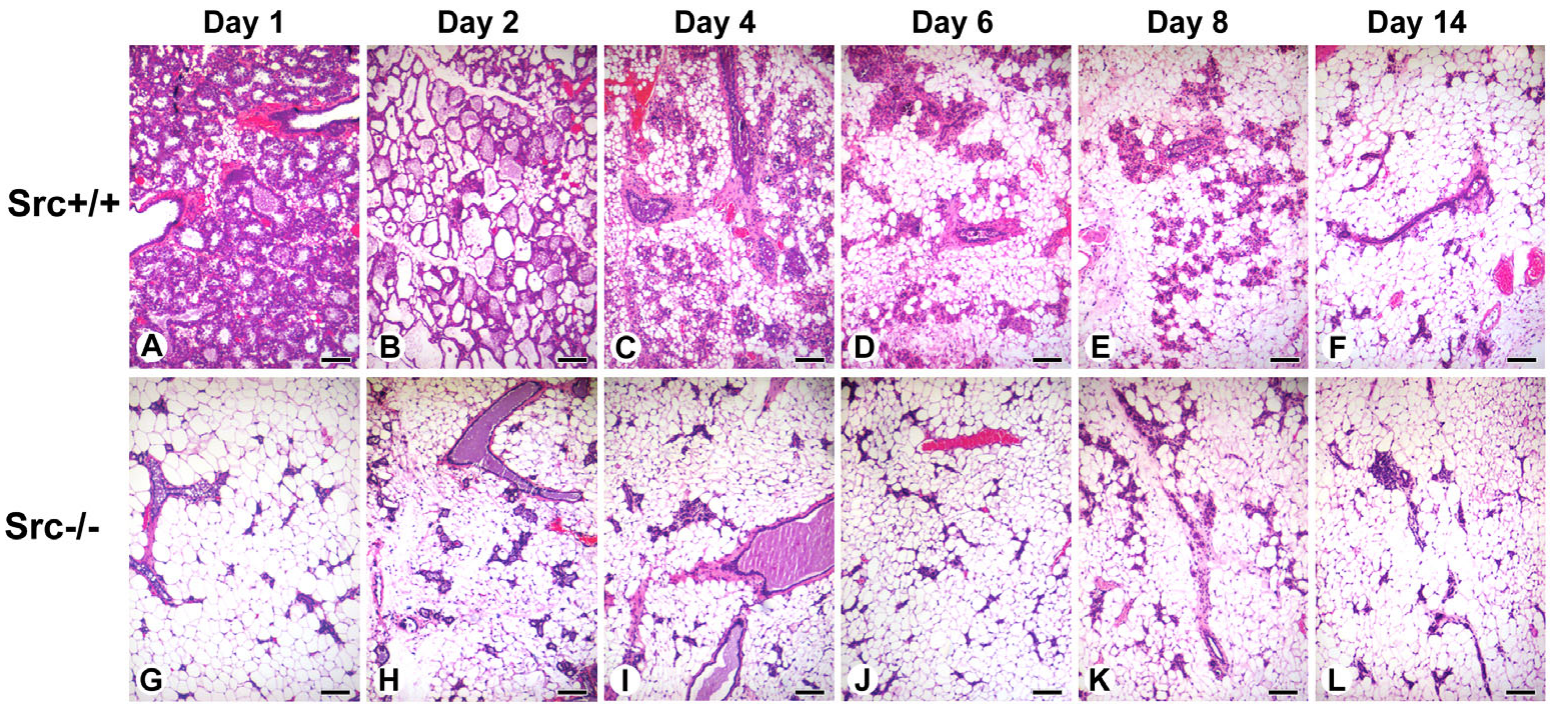
**Involution is accelerated in mammary glands from Src-/- mice**. Histological sections from the mammary glands of Src+/+ (A-F) and Src-/- (G-L) mice are shown at I 1 (A, G), I2 (B, H), I4 (C, I), I6 (D, J), I8 (E, K) and I14 (F, L). Scale bars represent 250 μm.

Following weaning of the pups, the mammary gland undergoes involution; a process during which approximately 80% of the mammary epithelial cells present in the lactating mammary gland undergo apoptosis and the entire mammary gland is remodeled back to a pubertal-like morphology [47,48]. Due to the difficulty of timing specific biochemical events during natural involution, involution is experimentally induced by withdrawal of pups from a lactating dam. Although involution induced by pup withdrawal results in a process that is significantly different on the molecular level from that which occurs following natural weaning [49], it does result in a series of reproducible and well-defined histological and biochemical changes [47,49]. Pups were withdrawn after L9 from Src+/+ and Src-/- dams to induce involution. The first day following pup withdrawal is referred to involution day 1 (I1). Twenty four hours after pup withdrawal (I1), secretory alveoli were still present in the mammary glands of Src+/+ mice (Figure 9, panel A). By I2 secretory alveoli were distended and mammary adipocytes could be observed in the Src+/+ mice (Figure 9, panel B). Collapse of the lobular alveolar units, numerous mammary adipocytes, and distended ducts were clearly present in the mammary glands of control Src+/+ mice by I4 (Figure 9, panel C). The remodeling of the mammary gland continued after this point and was largely complete by I8 in the Src+/+ mice (Figure 5, panel E). In contrast the sequence of events observed in the mammary glands of Src-/- mice was distinctly different; the histology of the mammary gland twenty four hours after pup withdrawal resembled the morphology of the I8 mammary gland in Src+/+ mice (Figure 9, compare panel G with panel E). Thus it appears that involution is dramatically accelerated in Src-/- mice, and may reflect the apparent failed secretory activation and the precocious involution that appears to occur in Src-/- dams even when pups are suckling and should be providing a stimulus for lactation and survival of mammary epithelial cells (see Figure 4, panels D and E).

**Figure 10:**
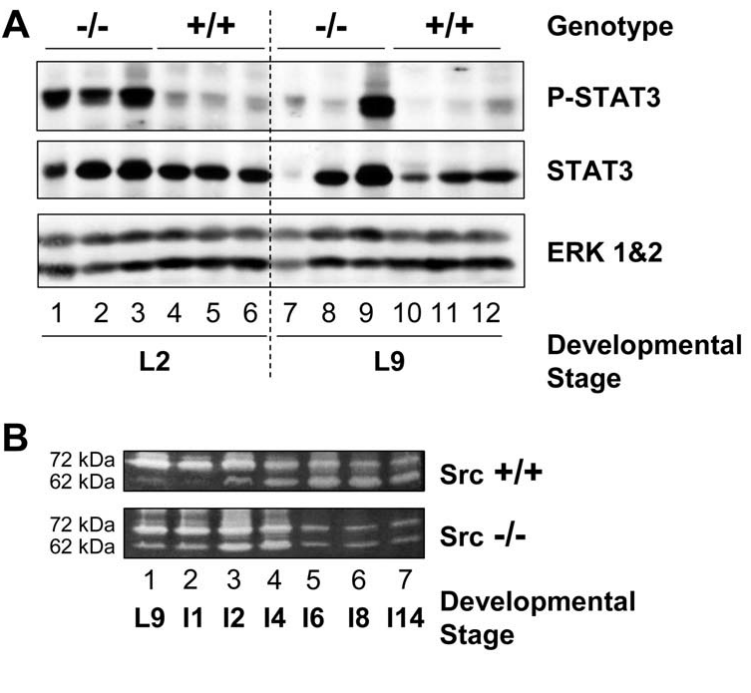
**Precocious activation of STAT3 and activation of MMP2 suggest early onset of involution**. A) The number 4 mammary gland was removed from Src-/- and Src+/+ mice at P18 (lanes 1–6), L2 (lanes 7–12) and L9 (lanes 13–18). Three separate mice were used per genotype and developmental stage. Protein lysates were prepared as described in the Materials and Methods sections, and immunoblotting conducted to detect phosphorylated STAT3 (tyrosine7^705^) (top panel), the total amount of STAT3 expression (middle panel), and the amount of ERK 1&2 as a loading control (bottom panel). B) Mammary gland lysates were prepared from Src+/+ (top panel) and Src-/- (bottom panel) mice at L9. I1, I2, I4, I6, I8, and I14. The positions of the 72 kDa molecular weight proenzyme and 62 kDa molecular weight active enzyme are indicated on the left side of the panels.

There are many biochemical markers that define mammary gland involution including activation of STAT3 [50], changes in the expression of Bcl2 family members [51-53], and activation of matrix metalloproteases [54]. We examined the expression and phosphorylation/activation of STAT3 as a marker of involution since STAT3 is activated early during involution and conditional tissue-specific deletion of STAT3 results in delayed involution [50,55]. Activation of STAT3 was detected in all three of the Src knockout mammary gland lysates isolated on day two of lactation (Figure 10A top panel, lanes 1–3), and one of the three Src-/- mice analyzed on day L9 (Figure 10A, lane 9). There was no phosphorylation/activation of STAT3 on either L2 or L9 in the Src+/+ mice, even though the total levels of STAT3 expression were comparable to those in the Src-/- mice (Figure 10A, top panel, lanes 4–6 and middle panel, compare lanes 4–6 to lanes 1–3). These data provide additional evidence for precocious involution occurring in the Src-/- mice, perhaps as a result of the failed secretory activation described above.

As a second marker for involution, we examined the activation of matrix metalloproteases in the mammary glands of both Src+/+ and Src-/- mice using a gelatinase assay in which the ability of MMP2 to degrade gelatin in a substrate gel is examined. Bands with molecular weights of 72 kDa and 62 kDa were observed (Figure 10B); the 72 kDa molecular weight band corresponds to the proenzyme of MMP2 and the 62 kDa molecular weight band corresponds to the activated form of MMP2. Consistent with previous results, the 62 kDa molecular weight band can start to be detected at I2 and is prominent by I4, I6 and I8 in the wild type mice (Figure 10B, top panel). In contrast, the 62 kDa molecular band was readily detected in mammary glands of Src-/- mice at L9 and I1, and was present at maximal levels by I2 and I4. (Figure 10B, bottom panel). Minimal levels of either form were present from I6 to I14 in the mammary gland from Src-/- mice. This provides further evidence that involution occurs precociously in the mammary glands of Src-/- mice.

## Discussion

The members of the Src family of protein tyrosine kinases are activated by a wide variety of stimuli including receptors for peptide growth factors, steroid hormone receptors, immune receptors, and integrin molecules. Because all of these receptors are important in different phases of mammary gland development, we were interested in exploring whether Src-like kinases mediated the downstream signaling events required for a functional mammary gland. Src, Fyn and Yes are widely expressed in different tissues, while the remaining family members (Blk, Hck, Fgr, Lyn, and Lck) are expressed in one or more lineages of hematopoietic cells [21,56]. We focused upon Src and Fyn in our studies because they are the major Src family members expressed at detectable levels during mammary gland development [33,57], and the low levels of the remaining Src family members could represent expression in infiltrating hematopoietic cells. Src-/- mice have osteopetrosis which results in thick bones and an altered shape to the skull. These changes result from a defect in bone-remodeling that is caused by nonfunctional osteoclasts [58]. Src-/- mice also lack teeth and were reported to be profoundly smaller by 10–12 days after birth [58]. Soriano *et al *reported that most of the mice die at 3–4 weeks if weaned, although a small number of mice could be maintained past five weeks if fed a soft diet [58]. Replacement of rodent chow with a liquid diet allows growth to a normal size and long-term survival.

**Figure 11:**
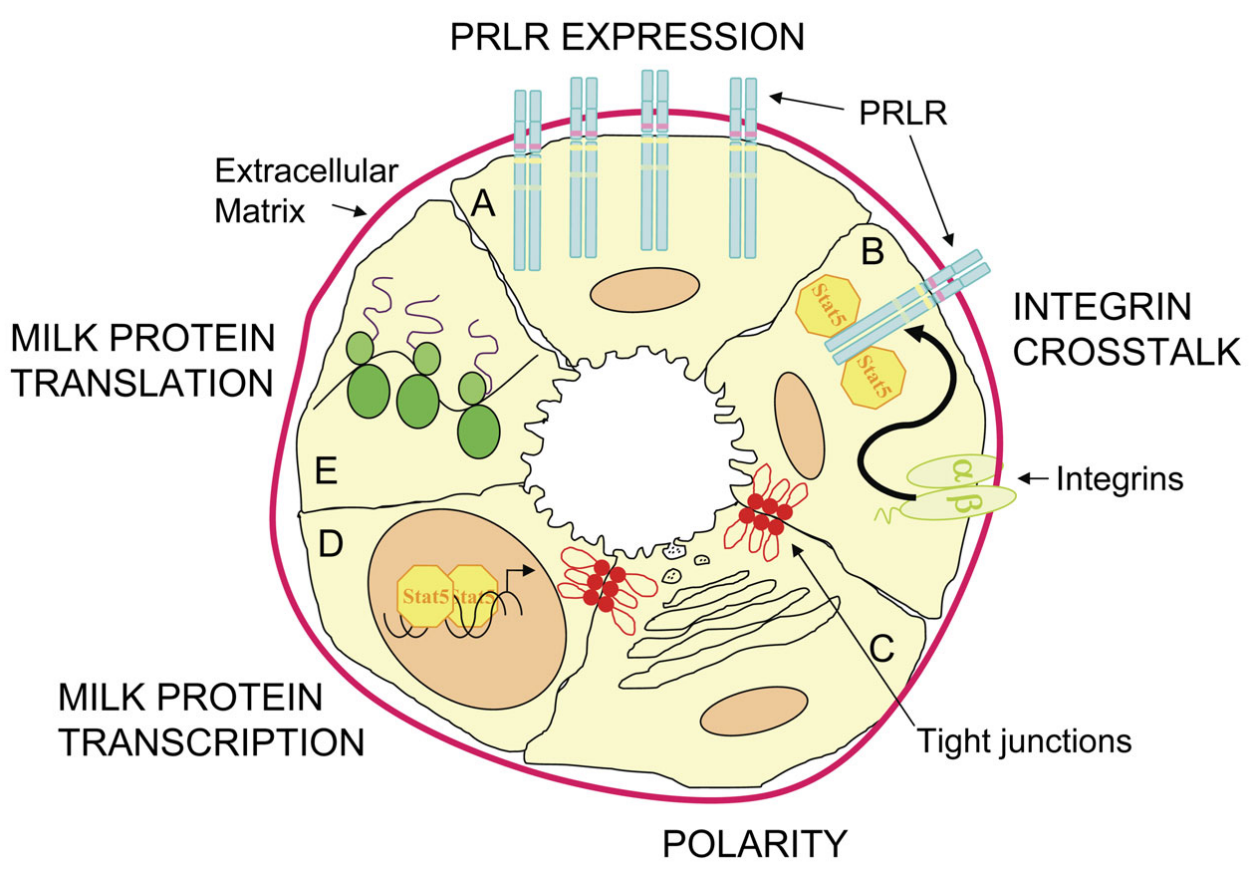
**Model of the pathways required for secretory activation and lactation that are perturbed in Src-/- mice**. A) PRLR EXPRESSION. In normal mice PRLR expression increases at parturition, this is absent in the Src-/- mice and they have decreased levels of both PRLR message and protein. B) INTEGRIN CROSSTALK. Integrin-mediated adhesion is essential for STAT5 activation downstream of PRLR signaling and Src may be a required mediator for the β 1 integrin-induced crosstalk. The Src-/- mice have diminished levels of STAT5 phosphorylation. C) POLARITY. Epithelial cell polarity is crucial for secretory activation. The mammary glands of Src-/- mice do not have organized lobuloalveolar structures and many of the epithelial cells do not show restricted apical staining suggesting that Src is required for epithelial cell polarity and organization. D) MILK PROTEIN TRANSCRIPTION. Src-/- mice have significantly less β-casein message compared to wildtype mice. This may be directly related to the decreased levels of STAT5 activation in these cells or Src may also be a required signaling molecule in the additional pathways necessary for milk protein transcription. E) MILK PROTEIN TRANSLATION. Milk protein expression is also regulated at the translational level. Src-/- mice have decreased levels of milk proteins and a possible role for Src in the post-transcriptional regulation has not been investigated.

We and others have described that Src is required for ductal elongation during puberty in Src-/- mice. Kim et al have suggested that this defect is due to diminished estrogen receptor signaling in the mammary gland [31], while we hypothesize that expression of Src is required in the stromal cell compartment (Richert et al, unpublished data). The data presented in this manuscript describe a novel role for Src in the mammary gland during secretory activation and/or lactation that was not previously anticipated by the phenotype of the Src knockout mice. We have observed that the growth of pups nursed by Src-/- dams is severely diminished and over 80% of the litters do not survive more than a few days. This phenotype is determined by the genotype of the dam and not the pups such that the growth of FVB, C57Bl6 and Src+/- pups nursed by Src-/- dams was diminished. The fact that over 90% of pups born to Src-/- mothers die within days, and that over 80% of fostered litters also die, indicates that a failed lactation is the predominant phenotype. The variation in the histology of the lactating mammary gland may reflect a physiological state in which survival of mammary epithelial cells is supported even when lactation is compromised. Analysis of the mammary gland of Src-/- mice during pregnancy and lactation reveals that large CLDs accumulate in the MECs of Src-/- mice following parturition providing evidence that secretory activation has not occurred [10]. Serum PRL levels in the Src-/- mice are normal indicating that neuronal stimulation of the hypothalamus, release of prolactin releasing factor, the response of the pituitary to this stimulus and hormonal signals from the pituitary are not affected by the absence of Src. We have demonstrated by gene expression profiling that expression of the long form of the PRLR increases at parturition in the mammary gland of normal mice [7,8], which is in agreement with other data using PCR-based approaches [42], however it appears that expression of the PRLR is diminished, although not absent, in the mammary glands of Src-/- mice (Figure 11). The decreased expression of the PRLR in the Src-/- mice can thus account for the diminished activation of the STAT5 transcription factor, which regulates expression of milk protein genes.

The impaired lactation we have observed in the Src-/- mice shares some features with those observed in the PRLR knockout mice described by Ormandy et al [59]. While is not surprising that the PRLR-/- mice exhibit severely diminished lobuloalveolar development during pregnancy and a complete failure of lactation, it was unexpected that young PRLR+/- mice also exhibit severely diminished lactation since these mice still have one functional PRLR allele. Lactation failure only occurs after the first pregnancy of young PRLR+/- mice however, and the success of lactation in PRLR+/- mice improves as the number of pregnancies increases or if the mice are older at the time of first pregnancy [59]. This implies that there is an important effect of the level of PRLR expression upon mammary gland development either late in pregnancy or during secretory activation. In the PRLR+/- mice this is manifested by the lactation failure at first pregnancy that is offset by further mammary gland development that occurs as the mouse ages such that older PRLR+/- mice successfully lactate. Thus the phenotype we have observed in the Src-/- mice may be directly related to the level of PRLR expressed at secretory activation.

As noted in the introduction, we have identified four potential regulatory genes whose expression dramatically increases at secretory activation: PRLR, Src, Akt, and SREBP1c [7,8]. In light of these observations, it is interesting to note that in the absence of Src, the increase in the expression of the long form of the PRLR at secretory activation does not occur in Src-/- mice as observed in wildtype mice, suggesting that Src is required for optimal PRLR expression in the mammary gland during the transition from pregnancy to lactation (Figure 11, panel A). Because the expected increase in PRLR expression and milk protein genes does not happen in the Src-/- mice we believe that secretory activation has not occurred. A recent study suggests that Akt1 plays a critical role in lactation, perhaps by regulating the levels and plasma membrane localization of GLUT1, as well as the levels of lipogenic enzymes whose expression are regulated by SREBP1 [60]. We have not examined whether the expression levels of either Akt or SREBP-1c are altered in the mammary gland of Src-/- mice.

As a means to confirm our *in vivo *observations, we chose to examine the differentiation of primary MECs established from Src+/+, Src+/-, and Src-/- mice. MECs were established from adult virgin mice following injection with progesterone and estradiol for 21 days to stimulate proliferation and differentiation of these cells. We used this approach because it allowed expansion of the epithelial cell compartment of the mammary gland in the absence of pregnancy which might have presented additional complications to the experimental design. MECs were plated on Matrigel, induced to differentiate following stimulation with PRL, and differentiation monitored by the expression of β-casein protein. At early time points after differentiation with PRL, the activation of STAT5 was lower in cultures of Src-/- MECs than in Src+/- or Src+/+ MECs, and the amount of β-casein protein was lower in Src-/- and Src+/- cells compared to Src+/+ MECs. There is an apparent dose-dependent effect on the amount of β-casein expressed in the cells with regard to the expression of Src. We have not detected a difference in the amount of PRLR in cultures of primary MECs from Src-/- mice compared to wildtype mice and thus suggest that there is an effect of Src upon either the transcription and/or translation of β-casein and by extension other milk protein genes (Figure 11, panels D and E).

A possible mechanism by which Src might regulate expression of β-casein is suggested by the observations of Streuli and his colleagues who have demonstrated a role for the cytoskeleton in PRL-dependent differentiation of MECs and that activation of STAT5 occurs in a integrin-dependent manner (Figure 11, panel B) [61-63]. Binding of integrins to extracellular matrix or peptide ligands leads to activation of focal adhesion kinase (FAK) [64-67]. Src is associated with FAK and Src phosphorylates FAK on at least one site that may regulate activation of downstream signaling molecules including phosphatidylinositol 3'-kinase [68-71]. In spite of the known association of Src with FAK and the ability of Src to phosphorylate FAK, we have not detected a decrease in tyrosine phosphorylation of FAK at site favored by Src in the Src-/- MECs implying that other tyrosine kinases are able to phosphorylate this site in these cells (H. Watkin and S.M. Anderson, data not shown). This also suggests that while Src may be an intermediate in the pathway by which integrins regulate milk protein gene expression, it is likely that the Src-mediated phosphorylation of FAK is not the means by which this occurs. The recent suggestion that Rac1 links integrins to lactational differentiation [62] causes us to wonder whether Src lies upstream, downstream, or is independent of Rac1.

The disorganized luminal structure of secretory alveoli in Src-/- mice also suggests an important role for Src in establishing the polarity of epithelial cells. Src family kinases have previously been implicated in both positive and negative regulation of epithelial tight junction assembly and polarization. Src-dependent phosphorylation of Par3 downstream of EGF receptor activation is required for tight junction formation [72]; however it has also been suggested that ErbB2-dependent activation of Src results in disruption of epithelial cell-cell contacts [73], thus highlighting the multiple roles of Src within a cell and the different effects that depend on upstream signals and cellular context. In the case of normal mammary gland development, it appears that Src plays a positive role with regard to the structural organization of the secretory epithelial cells within alveoli and the polarity of these cells (Figure 11, panel C).

Additionally, several links have been reported between the activity of Src kinase and Rho GTPases. The small GTPases of the Rho family regulate many cellular functions including cell adhesion, migration and cell-cell junctions [74]. Src is involved in the activation of Rac [75] and Cdc42 [76] and can inhibit Rho [77,78]. In the mammary gland Rac1 has been found to be required for lactational differentiation in a primary cell culture model [79]. Whilst we have not investigated the activation of Rac1 in the mammary glands of Src-/- mice this may be another pathway that could be perturbed by a lack of Src. PKN1 transgenic mice are another mouse model that display impaired secretory activation [80]. The expression of a constitutively activated form of the Rho effector protein PKN1 results in impaired tight junction sealing. This implies that Rho negatively regulates tight junction closure. The possible requirement for Src to down regulate Rho is an additional mechanism by which Src may regulate secretory activation. Whilst we have observed disorganized alveolar structures in the Src-/- mice we have not established which of the many pathways that regulate tight junction formation, cell polarization, and differentiation are perturbed by the lack of Src in mammary epithelial cells.

Secretory activation occurs at parturition and is required for lactation. While the mechanisms underlying secretory activation remain unknown, we have noted that there are four signaling molecules/transcription factors whose expression is increased at this time; PRLR, Src, Akt1, and SREBP1c [7,8]. Secretory activation requires the coordinate increase in the expression of milk protein genes as well as the genes that are required for the synthesis of the lipid component present in milk. When the pattern of expression of milk proteins genes is closely examined, it becomes apparent that there are actually two different classes of milk proteins genes; the level of the casein-like class increases over the course of pregnancy and then undergoes a further three-fold increase at secretory activation (α-casein, β-casein, δ-casein, κ-casein, whey acidic, and ADPH), while the second does not increase during pregnancy and the RNA level only increases at secretory activation (δ-casein, α-lactalbumin, MUC1, xanthine oxide dehydrogenase, and butyrophilin) [10]. The fact that we are able to detect β-casein RNA at P18 suggests to us that the increase in milk proteins genes in the early-expressed class, referred to as the casein-like genes above, occurs in the Src-/- mice, however the second increase in expression that occurs at secretory activation does not occur. This is re-enforced by the fact that there is no increase in the amount of β-casein RNA between P18 and L2 in the Src-/- as observed in the Src+/+ control mice. We have recently noted that expression of lipogenic enzymes increases dramatically at secretory activation [7,8], and it would be of great interest to determine whether expression of these genes is altered in Src-/- mice. The fact that large CLDs are observed in the mammary glands from Src-/- mice clearly indicates that lipid biosynthesis during pregnancy is normal; however, it is not clear whether it increases following parturition.

The mammary glands of Src-/- mice undergo precious involution, as demonstrated by histology and biochemical markers. The decisive trigger for the onset of involution and apoptosis of the epithelial cells within the mammary gland is unknown, however a number of molecules and stimuli have been shown to play a role. These include milk stasis, leukemia inhibitory factor, STAT3 activation, interleukin 6, Fas ligand, loss of integrin activation, transforming growth factor-β 3 and insulin-like growth factor-binding protein 5 [81-87]. Lactation failure has been observed in the mammary glands of several other transgenic mice, including models with impaired tight junction sealing and another in which the milk has a high fat content which may result in milk stasis due to the inability of pups to remove milk from the mammary gland [80,88]; in both of these models lactation failure leads to activation of a precocious involution program. The lactation failure observed in the Src-/- mice may result in a dominant signal that results in precocious involution and thus it is not possible to determine whether Src is required for survival of mammary epithelial cells. The fact that increased apoptosis was not observed in primary mammary epithelial cells from Src-/- mice, and the fact that mammary epithelial cells proliferate during pregnancy suggest that Src may not be required for cell survival although we have not directly tested this possibility.

Our studies indicate a new role for Src that had not been anticipated based upon previous studies of Src-/- mice, or the study of mammary gland development using either *in vivo *or *in vitro *systems. The role for Src in secretory activation and lactation is apparently unique to Src as we have not observed this phenotype in Fyn-/- mice. The lactation failure in the Src-/- mice demonstrates that other Src family members are not able to compensate for the loss of Src. Given the number of different regulatory pathways activated in secretory activation, which includes both Akt1 and SREBP1, it will be of great interest to determine whether expression of any of the regulatory molecules that lie downstream of the PRLR is able to compensate for the loss of Src in secretory activation. We would anticipate that expression of some might complement the loss of Src in stimulating expression of milk protein genes in MECs, while other molecules might be required to complement the loss of Src in vivo. Expression of constitutively activated signaling molecules in vitro and in the mammary gland in vivo, via either the use of recombinant human adenoviruses or expression of transgenes in the mammary gland will provide additional insights into the multiple actions of Src in this tissue.

## Conclusion

Src is essential in the mammary gland for successful lactation as Src-/- mice are unable to support the growth, and often the survival, of their pups. Histological analysis of mammary glands from late pregnant and post-partum Src-/- mice suggests that Src-/- mice fail to undergo secretory activation. The precise mechanisms by which secretory activation are controlled are not yet fully understood, however we have studied some of the known critical factors in order to investigate the role of Src, summarized in Figure 11. Src is required for an increase in PRLR expression and may also be required for the crosstalk from integrin signaling that is essential for signal propagation down-stream of the PRLR (Figure 11, panels A and B). Tight junction closure is a hallmark of secretory activation and epithelial polarity is essential for lactation. The disorganized luminal structure of secretory alveoli in Src-/- mice suggest an important role for Src in establishing the polarity of epithelial cells (Figure 11, panel C). Src-/- mice also have decreased levels of β-casein message (Figure 11, panels D and E). *In vitro *studies conducted on MEC from Src-/- mice suggest a primary role for Src in regulating either the transcription or translation of milk protein genes that may be distinct from its role in secretory activation.

## Methods

### Mice

Src-/- [58] mice were obtained from Dr. Paul Stein (Northwestern University School of Medicine, Chicago, IL). The knockout line is on a mixed 129Sv × C57Bl6 background, with approximately 75% of the alleles being of C57Bl6 (Personal communication, Paul Stein). Thus Src-/- and Src+/- mice are mixed C57Bl6 and 129Sv. The Src-/- mice lack teeth [58] and therefore were fed Liquid Rat Diet (Bio-Serv, Frenchtown, NJ; Product #F1259SP) and water *ad librium*. All mice were maintained on a normal 12-hour day/night cycle. Control, Src+/+ F1(C57Bl6 × 129Sv) and F2 [C57Bl × F1(C57Bl6 × 129Sv)] mice were either obtained from The Jackson Laboratory (Bar Harbor, ME), or were generated in-house. All mice were maintained in the Animal Care Facilities at the University of Colorado Health Sciences Center, an AAALAC-approved facility, and used in accordance with institutional ACUC-approved protocols. DNA was extracted from 1.5 cm sections of tails using the Puregene DNA Purification Kit (Gentra Systems, Minneapolis, MN) according to the manufacturer's directions or using a protocol described previously [89], with the modification that one phenol:chloroform:isoamyl alcohol (25:24:1) extraction was performed prior to ethanol precipitation. DNA pellet was resuspended in TE (10 mM Tris, pH 8.0, 1 mM EDTA). Genotyping of Src-/- mice utilized the following primers: NeoSrc, 5'-TCATAGCCGAATAGCCTCTCCAC-3'; forward Src primer, 5'-AGCAACAAGAGCAAGCCCAAGGAC-3', and reverse Src primer, 5'-GTGACGGTGTCCGAGGAGTTGAAG-3'. One microgram of DNA was amplified by PCR using primers (50 pmol/reaction) described above: 95°C for 10 min; 30 cycles of 94°C for 1.5 min, 60°C for 1.5 min, and 72°C for 1.5 min; followed by a 10 min extension time at 72°C. This reaction produces a 200 base pair product from the wildtype Src allele and a 400 base pair fragment from the rearranged Src allele.

### Immunoblot analysis

The fourth inguinal mammary gland was removed at the indicated developmental stage and snap frozen in liquid nitrogen. The gland was ground in liquid nitrogen with a pestle and mortar then proteins were extracted by homogenizing in lysis buffer [(50 mM Tris pH 7.4, 150 mM NaCl, 2 mM EDTA, 50 mM NaF, 1% Triton X-100, 1% DOC, 0.1% SDS, 1 mM DTT, 5 mM sodium orthovanadate, 100 μg/ml PMSF, and a protease inhibitor cocktail (Sigma # P 8340, St. Louis, MO)]. Homogenates were centrifuged at 13,000 × g for 30 min at 4°C. Soluble fractions were stored at -80°C. Protein assays were performed using the Pierce Coomassie Plus protein assay reagent (Pierce Chemical Company, Rockford, IL). 50–100 μg amounts of total protein were resolved on SDS-polyacrylamide gels, transferred to polyvinylidene difluoride (PVDF) membrane (Immobilon, Millipore, Bedford, MA), and immunoblotted with the desired antibody. Bound antibodies were detected using ECL according to manufacturer's recommendations (Western Lighting Perkin Elmer, Boston, MA). Anti-Src monoclonal antibodies 327 and 2–17 were prepared in house. Rabbit anti-STAT3 (SC-482), rabbit anti-ERK (SC-93), rabbit anti-STAT5A (SC-1081), rabbit anti-Raf (SC-133), rabbit anti-prolactin receptor (SC-20992), and HRP-conjugated rabbit anti-actin (SC-1616) antibodies were obtained from Santa Cruz Biotechnology (Santa Cruz, CA). Rabbit anti-phospho-Src (Tyrosine^416^; Cat # 2101), rabbit anti-phospho-STAT5 (Tyrosine^694^; Cat # 9351), and rabbit anti-phospho-STAT3 (Tyrosine^705^; Cat#9131) antibodies were obtained from Cell Signaling Technology (Beverly, MA). A rabbit polyclonal antiserum raised against mouse milk-specific proteins (Cat # YNRMTM) was obtained from Accurate Chemical and Scientific Corp. A rabbit anti β-casein antibody was obtained from Margaret C. Neville (University of Colorado Health Sciences Center). HRP-conjugated Anti-rabbit and Anti-mouse secondary antibodies were from Bio-Rad (Cat # 170-6515 and Cat # 170-6516 respectively).

### Preparation of mammary gland whole-mounts and thin sections for histological evaluation

The second and third thoracic and fourth inguinal mammary glands of control and transgenic mice were dissected on days 6, 12, and 18 of pregnancy (P6, P12, P18); days 2 and 9 of lactation (L2, L9); and days 1, 2, 4, 6, 8, and 14 of involution (I1, I2, I4, I6, I8, I14). Pregnancy time points indicate the number of days after the formation of vaginal plugs upon mating. Lactation time points imply the number of days the female lactated after parturition (postpartum days), and the number of pups per litter was normalized to eight by the addition of same-age pups from control litters. For involution time points, pups were removed on the 9^th ^day postpartum to induce forced involution.

Whole-mount analysis of dissected mammary glands was preformed as described previously [57,90,91].

For histological analysis, dissected mammary glands were fixed in 10% neutral buffered formalin, embedded in paraffin, sectioned (4 μm), and stained with hematoxylin and eosin. Histological sectioning and staining was performed by the Histology Service, Department of Pathology, University of Colorado School of Medicine.

### Immunofluorescence and lipid droplet analysis

Mammary glands were dissected at the indicated developmental stages, fixed in 10% neutral buffered formalin, and embedded in paraffin. Sections were cut at 5 microns. Following dehydration with graded alcohols, microwave antigen retrieval was performed for 20 min in 10 mM sodium citrate, pH 6.0. The sections were permeabilized with 0.2% Triton X-100 in PBS, blocked with 5% goat serum in PBS, and incubated with a rabbit polyclonal anti-adipophilin antibody (ADRP; also known as adipocyte differentiation-regulated protein) (Dr. Thomas Keenan, Virginia Polytechnic Institute and State University, Blacksburg, VA) at a dilution of 1:200. The sections were then incubated with Cy3-conjugated anti-rabbit IgG (Jackson ImmunoReasearch Laboratories, Inc, West Grove, PA) at a 1:150 dilution, Oregon-Green 488-conjugated wheat germ agglutinin (WGA) (Molecular Probes, Eugene, OR) at a 1:250 dilution and 0.6 μg/ml DAPI (Sigma). Images were collected using SlideBook software (Intelligent Imaging Innovations, Inc, Denver, CO) on a Nikon Diaphot TMD microscope equipped for fluorescence with a Xenon lamp and filter wheels (Shutter Instruments, Novato, CA), cooled CCD camera (Cooke, Tonawanda, NY) and stepper motor (Intelligent Imaging Innovations, Inc).

### Quantitation of serum prolactin levels

Blood was collected from five Src-/-, five Src+/-, and five Src+/+ mice on day seventeen of pregnancy and day two of lactation, and plasma prepared. Quantitation of prolactin levels in the plasma was conducted using a radio-immune assay by Dr. A. F. Parlow of the National Hormone and Peptide Program, UCLA Medical Center, Torrance, CA.

### Measurement of Pup Weights

As a means to assess the efficiency of lactation in Src-/- dams the weights of fostered pups nursed by Src -/- dams were compared to the weights of pups nursed by control FVB, C57Bl6 and Src+/- dams. Litters were normalized to 8 pups and the whole litter was weighed daily. The average pup weight was calculated by dividing the whole litter weight by the number of pups weighed (eight). The litters of the Src-/- dams were replaced with control FVB pups of the same age on the first day postpartum and fostered pup weights were recorded for 9 days of lactation. In addition to fostering new born litters older, more experienced C57Bl6 or Src+/- pups were also used to assess lactational competency. For the control experiments litters of three individual dams were recorded and the error bars depict the s.e.m. of the average pup weight in each of those control litters.

### Quantitative RT-PCR analysis

The fourth inguinal mammary gland was removed at the developmental stage indicated and stored in RNA later (QIAGEN) according to the protocol. Total RNA was prepared using the RNeasy Mini Kit (QIAGEN) and RNA integrity analyzed by RNA 6000 Nano Assay (Agilent Technologies, Palo Alto, CA). 1 μg RNA was reverse transcribed using a mix of random hexamers and oligo dT primers and SuperScript III RT (Invitrogen). PCR primer and probe sets were designed using TaqMan Primer Express™ software to target a splice boundary within a message sequence to avoid the amplification of genomic DNA. All primers, labeled probes and synthetic amplicon sequences were purchased from either Applied Biosystems or Integrated DNA Technologies. RT-PCRs were performed using an Opticon Monitor II RT-PCR detection system plus the accompanying software version 2.02.24 (MJ Research, Bio-Rad Laboratories, Inc., Hercules, CA). Copy numbers of the investigated genes were calculated from the respective standard curves, which were generated with amplicons, using the formula 10^[(Ct-Y intercept)/(slope)]. For each sample the ratio of the copy number of the investigated gene to the copy number of GAPDH produced normalized data. Welch's *t *test was used to evaluate the statistical significance (defined as *P *< 0.05).

### Preparation of primary mammary epithelial cells and in vitro differentiation

Mammary epithelial cells (MECs) are traditionally prepared from the mammary glands of four to six late pregnant mice that are 10–12 weeks old. Because of the difficulty in obtaining multiple Src-/- mice that were impregnated within a narrow window of time to allow the preparation of MECs from late pregnant mice, we chose to inject eight- to ten-week old virgin Src+/+, Src+/-, and Src-/- mice with 1 mg progesterone and 1 μg estradiol daily for 21 days, six mice of each genotype were used for each preparation of MECs. All of the mammary glands were removed, and the surrounding tissue and inguinal lymph nodes removed to minimize contamination of the cultures. MECs were prepared as described by Pullan and Strueli [92]. The isolated aggregates of mammary organoids were washed 3× in F12 medium to remove single cells such as fibroblasts. The primary MECs were cultured on Matrigel-coated dishes in growth medium (50% DMEM with glutamine, 50% F12 with glutamine, 10 mM HEPES, 10% Fetal calf serum, 5 μg/ml insulin, 1 μg/ml hydrocortisone, and 10 ng/ml recombinant epidermal growth factor with penicillin and streptomycin), for 72 hours. Differentiation of MECs was induced by changing the culture medium to differentiation medium (DMEM with glutamine, 10 mM HEPES, 5 μg/ml insulin, 1 μg/ml hydrocortisone, with penicillin and streptomycin). The cells were cultured in this medium in the presence or absence of 3 μg/ml ovine prolactin for 24 or 72 hours before lysis in 2 × NET buffer (100 mM Tris pH 7.4, 300 mM NaCl, 10 mM EDTA, 2% Nonidet^®^-P40, supplemented with freshly added phosphatase inhibitors 100 mM NaF, 10 mM sodium orthovanadate, and a protease inhibitor cocktail (Roche Diagnostics Gmbh Complete EDTA-free cat # 11 873). Homogenates were centrifuged at 13,000 × g for 30 min at 4°C and immunoblot analysis performed as described above.

### Zymogram gels

The samples to be analyzed were prepared from frozen tissue in mammary gland extraction buffer and protein concentrations quantified as described above. 30 μg of total protein was diluted in SDS sample buffer lacking β-mercaptoethanol and was not heat denatured prior to loading. Samples were separated on a 7.5% SDS gel containing 3 mg/ml of type A porcine gelatin (Sigma Aldrich, cat. #G-2500). The gels were run at 60 V for 30–45 min through the stacking gel and then at 120 V until the samples reached the bottom of the running gel. The gels were washed twice for 30 minutes in 2.5% Triton X-100 at room temperature while rocking. The gels were then incubated on a rocking platform at 37°C for 24–72 hours in substrate buffer (50 mM Tris-HCl, pH 8.0; 5 mM CaCl_2_). The gels were stained for one hour at room temperature with 0.25% Coomassie Brilliant Blue-R, and destained overnight in water.

## Abbreviations

ADPH, adipophilin; CLD, cytoplasmic lipid droplet; DAPI, 4'-6-diamidino-2-phenylindole; FAK, focal adhesion kinase; MECs, mammary epithelial cells; MMP, matrix metalloprotease; MRNA, messenger RNA; PRL, prolactin; PRLR, prolactin receptor; QRT-PCR, quantitative real time polymerase chain reaction; RNA, Ribonucleic Acid; SREBP, sterol response element binding protein; STAT3, signal transducer and activator of transcription-3; STAT5, signal transducer and activator of transcription-5; WGA, wheat germ agglutinin.

## Authors' contributions

HW carried out the pup weight experiments, quantitative RT-PCR, primary cell culture, western blot analysis and drafted the manuscript. MR and AL prepared the histological sections. MR revised the manuscript. AL managed the mouse colonies. KT did the zymogram gels. JM performed the staining for figure 5. SA conceived of the study, and participated in its design and coordination, and helped to draft the manuscript. All authors read and approved the final manuscript.
